# *Klf4* protects thymus integrity during late pregnancy

**DOI:** 10.3389/fimmu.2023.1016378

**Published:** 2023-04-27

**Authors:** Lucyle Depoërs, Maude Dumont-Lagacé, Vincent Quoc-Huy Trinh, Chloé Houques, Caroline Côté, Jean-David Larouche, Sylvie Brochu, Claude Perreault

**Affiliations:** ^1^ Department of Medicine, Institute for Research in Immunology and Cancer, Université de Montréal, Montréal, QC, Canada; ^2^ ExCellThera, Inc., Montréal, QC, Canada; ^3^ Piercing Star Technologies, Rabat, Morocco; ^4^ Department of Pathology and Cellular Biology, Institute for Research in Immunology and Cancer, and Centre de recherche du Centre hospitalier de l’Université de Montréal, Université de Montréal, Montréal, QC, Canada; ^5^ Institut de Génétique Moléculaire de Montpellier, Université de Montpellier, Montpellier, France

**Keywords:** thymus, thymic epithelial cell, pregnancy, thymic regeneration, degeneration, thymic involution, Klf4

## Abstract

Pregnancy causes abrupt thymic atrophy. This atrophy is characterized by a severe decrease in the number of all thymocyte subsets and qualitative (but not quantitative) changes in thymic epithelial cells (TECs). Pregnancy-related thymic involution is triggered by progesterone-induced functional changes affecting mainly cortical TECs (cTECs). Remarkably, this severe involution is rapidly corrected following parturition. We postulated that understanding the mechanisms of pregnancy-related thymic changes could provide novel insights into signaling pathways regulating TEC function. When we analyzed genes whose expression in TECs was modified during late pregnancy, we found a strong enrichment in genes bearing KLF4 transcription factor binding motifs. We, therefore, engineered a *Psmb11-iCre : Klf4^lox/lox^* mouse model to study the impact of TEC-specific *Klf4* deletion in steady-state conditions and during late pregnancy. Under steady-state conditions, *Klf4* deletion had a minimal effect on TEC subsets and did not affect thymic architecture. However, pregnancy-induced thymic involution was much more pronounced in pregnant females lacking *Klf4* expression in TECs. These mice displayed a substantial ablation of TECs with a more pronounced loss of thymocytes. Transcriptomic and phenotypic analyses of *Klf4* ^-/-^ TECs revealed that *Klf4* maintains cTEC numbers by supporting cell survival and preventing epithelial-to-mesenchymal plasticity during late pregnancy. We conclude that *Klf4* is essential for preserving TEC’s integrity and mitigating thymic involution during late pregnancy.

## Introduction

The thymus is the sole organ that can produce classic adaptive T cells (1). Its structure and function are remarkably conserved in vertebrates (2). No other organ can compensate for impaired thymic function (3). This is problematic, considering that thymic function correlates with life expectancy and that progressive thymus atrophy affects all aging subjects (4–7). Furthermore, T cells generated extrathymically in transgenic mouse models are autoreactive and fail to protect against pathogens (3, 8). Thymic epithelial cells (TECs) are responsible for the unique ability of the thymus to generate a functional T-cell repertoire (9, 10).

Acute thymic involution can be triggered by several drugs and pathogens (11). Notably, pregnancy is a situation where thymic involution plays a physiological role since it is necessary for normal fertility (12). Pregnancy leads to a marked decrease in thymic weight and thymocyte numbers beginning during the second trimester and culminating in the third trimester (13, 14). Thymocyte loss during pregnancy is not associated with reduced TEC numbers but depends on functional changes in TECs, particularly the cTEC subset (15). This is coherent with the fact that this atrophy is caused by progesterone and that cTECs upregulate the expression of nuclear progesterone receptors during pregnancy (16–18). TEC-specific deletion of the nuclear progesterone receptor prevents thymic involution but reduces fertility (18). Pregnancy-related thymic involution is severe but remarkably transient as thymic cellularity is rapidly restored following parturition (15). Thus, with this great amplitude and well-orchestrated kinetics, pregnancy-associated thymic involution is an attractive model for identifying regulators of TEC function and homeostasis.

To identify transcription factors (TF) regulating TEC function and homeostasis during late pregnancy, we analyzed the transcriptome of TECs harvested at different time points from the end of gestation to D28 post-delivery. We found a substantial enrichment of KLF4 binding motifs in genes differentially expressed in TECs from pregnant females. *Krüppel-like factor 4* (*Klf4*) is an evolutionarily conserved zinc finger-containing transcription factor highly expressed in the epithelium of various tissues, including the lung, intestine, and skin (19–22). It is one of the four classic factors instrumental in the induction of pluripotent stem cells from somatic cells (23). *Klf4* exerts essential roles in several cellular processes, such as cell cycle, cell survival, and differentiation (24). However, the *Klf4* function is cell-type and context-specific and can show opposite effects under certain conditions (24). For instance, *Klf4* inhibits the proliferation of the intestinal epithelium following irradiation and oppositely exerts a pro-proliferative activity during the regenerative phase (25). More importantly, *Klf4* has been described as a protector of epithelium integrity (26). Thus, *Klf4* null mice die within 15 h after birth due to defects in skin barrier formation (27).

The present study aimed to evaluate the potential role of *Klf4* in TEC biology. We found that KLF4 was expressed at high levels in TECs of non-pregnant females and during late pregnancy. While *Klf4* transcription decreased abruptly during the early phase of post-partum thymic regeneration, KLF4 protein is highly stable. Although the deletion of *Klf4* in TECs induced only minor thymic changes in homeostatic conditions, its absence dramatically altered thymic cellularity during late pregnancy. Transcriptomic and phenotypic analysis revealed that this phenomenon was due to 1) reduced survival of cTECs, and 2) acquisition of mesenchymal-like features in cTECs. Finally, exacerbated thymic involution in *Klf4*-deficient females showed long-lasting effects on post-partum thymic regeneration. Our results show a critical role for *Klf4* in maintaining cTEC integrity during late pregnancy.

## Materials and methods

### Mice

C57BL/6J mice were purchased from The Jackson Laboratory (JAX stock #000664). B6.129S6-Klf4^tm1Khk^/Mmmh (*Klf4^lox/lox^*) mice were obtained from Mutant Mouse Resource and Research Center [MMRRC:029877-MU; (28)]. Psmb11-iCre knock-in mice were kindly provided by Dr. Yousuke Takahama (29). *Psmb11^iCre/WT:^ Klf4^lox/lox^* (KO) mice used in this study were obtained by mating *Klf4^lox/lox^* and Psmb11-iCre parents. *Psmb11^WT/WT:^Klf4^lox/lox^* littermates (LOX) were used as controls. Genomic DNA PCR further confirmed the specificity of *Klf4* deletion in TECs. Successful deletion of *Klf4^lox/lox^* resulted in a 425-bp band, while the *Klf4^lox/lox^* allele without deletion gave a 296-bp product. To study the thymus during late pregnancy and at D16 and D28 post-partum, we mated KO or LOX females with C57BL/6J males for 72 hours. We analyzed the thymus of pregnant females 18 days following the first day of mating. Consequently, pregnant females were studied in the third trimester (16-18 days) of pregnancy. As lactation causes a delay in post-partum thymic regeneration, pups were removed at birth. Unless indicated otherwise, 10 to 14 weeks aged females were used. All mice were group-housed and maintained under specific pathogen-free conditions at the Institute of Research in Immunology and Cancer. All procedures were in accordance with the regulations of the Canadian Council on Animal Care guidelines and approved by the Comité de Déontologie de l’Expérimentation sur les Animaux de l’Université de Montréal.

### Flow cytometry and cell sorting

In this study, TECs were enriched as previously described (14). Briefly, thymi were mechanically disrupted and enzymatically digested with DNase I (Sigma-Aldrich), papain (Worthington-Biochem), and collagenase D (Sigma-Aldrich). To compare epithelial cells from the thymus, lung, colon, and skin and to quantify thymic fibroblasts (tFbs), we harvested cells by dissociating tissues with a mix of DNase and Liberase (15, 30). Thymocytes were extracted from the thymus by mechanical force. Single-cell suspensions were stained with appropriate antibodies before subsequent analyses. The list of antibodies used for flow cytometry analyses can be found in **Supplementary Table 1**. Throughout the paper, TECs are defined as EpCAM^+^CD45^−^, while the cTEC and mTEC subsets were defined as UEA1^–^ and UEA1^+^ TECs, respectively. Flow cytometry was performed on a ZE5 (Bio-Rad) apparatus or a Canto flow cytometer (BD Biosciences), and cell sorting was performed using a three-laser FACSAria (BD Biosciences). Data were analyzed using the FACSDiva or FlowJo software.

### RNA sequencing

Poly-A enriched mRNA sequencing (RNA-seq) was performed on cell-sorted epithelial cells after enzymatic digestion of the tissues. RNA was extracted using TRIzol™ (Life technologies) from three replicates per genotype, and three mice were merged per replicate to obtain a range of cell numbers between 23 700 and 100 000. RNA-extracted samples were purified using the RNeasy Micro kit (Qiagen) following the manufacturer’s instructions. Total RNA was quality ascertained using the Agilent Bioanalyzer RNA Pico. Transcriptome libraries were generated using the KAPA RNA HyperPrep PolyA (Roche). Single-end sequencing was performed with the Nextseq500 Illumina sequencer (1 x 75 nt). Sequencing adapters and 3’ bases of low quality were removed using Trimmomatic version 0.35. Sequences were aligned to the mouse reference genome GRCm38 (or mm10) using STAR version 2.5.1b. Gene expression quantification was computed with RSEM and Kallisto (version 0.46.0) in transcript per million (TPM) and count per million (cpm), respectively. We also performed analyses on previously published TEC RNA-seq data (GEO: GSE 138494, (15)) and publicly available single-cell TEC RNA-seq data (ArrayExpress: E-MTAB-8560, (31)). Reads were pseudo-aligned using Kallisto on the murine reference genome (mm10). Read count normalization, log transformation, dimensionality reduction, and data visualization were performed using the scatter (version 1.18.16) and scran (version 1.18.7) packages.

### Immunohistology and immunofluorescence

Thymi were fixed in 10% formalin, embedded in paraffin, and sliced into 4-μm-thick sections. Central sections were stained with H&E and scanned with a NanoZoomer Digital Pathology system (Hamamatsu) with a magnification of 40X. We analyzed the images using the NDP.view software (version 2.7.52, Hamamatsu). For immunostaining, sections were de-paraffinized, rehydrated, and treated with BOND Epitope Retrieval Solution 2 (an EDTA-based pH 9 epitope retrieval solution) in Bond RX Stainer (Leica Biosystems, Buffalo Grove, IL, USA). Sections were stained with primary antibodies, followed by staining with secondary antibodies and DAPI (Life Technologies). A wash step with PBS preceded all staining steps. The omission of primary antibodies and their replacement by isotype were used as controls. Specifications of primary and secondary antibodies are described in **Supplementary Table 1**. Klf4 images were acquired with a Leica SP8 confocal microscope (Leica Microsystems, Wetzlar, Germany) with 40X and 63X objectives and analyzed using LAS-X (Leica Microsystems, Wetzlar, Germany) software. Cell populations defined by K8, K5, VIM, and PDPN staining were quantified using the software QuPath (version 0.3.2; Queen’s University Belfast; Northern Ireland). Eighteen images with x20 magnification of the cortex, the cortico-medullary junction (CMJ), and the medulla were acquired from two KO and LOX mice. 8-bit channel-based images were exported and stacked in Fiji [(http://fiji.sc/ (32)]. VIM-K8 and PDPN-K8-K5 co-expression was assessed by performing the QuPath cell detection algorithm based on the DAPI channel, and automated quantification of the underlying staining intensity of VIM, PDPN, K5, and K8. The Create Single Measurement Classifier function was used to estimate the classification thresholds with the Live Preview function. A pathologist (VQ-HT) correlated automated positive cell classification with the marker staining in a control LOX mouse. This classifier was then applied blindly to all control and experimental mice.

### EdU administration

Mice received a single intraperitoneal injection with 100 μl of 1 mg/ml 5-Ethynyl-2’-deoxyuridine (EdU) in PBS on the first day of treatment. Mice subsequently received drinking water containing 0.3 mg/ml EdU for 12 days. Drinking water was replaced by freshly prepared EdU every three days. EdU uptake in TECs was detected by flow cytometry using an EdU Flow Kit (Thermofisher) following the manufacturer’s instructions (see **Supplementary Table 1**).

### Identification and analysis of differentially expressed genes (DEGs)

DEGs between KO and LOX females were identified using the limma package with the voom function and the treat method in R software with thresholds of FC ≥ 1.5 and *p*-value ≤ 0.05. We only kept genes with expression higher than one cpm in at least three samples. Gene ontology (GO) enrichment analysis was performed using g:Profiler (version e106_eg53_p16_65fcd97) with g:SCS multiple testing correction method applying a significance threshold of 0.05 [https://biit.cs.ut.ee/gprofiler/gost, (33)].

Gene Set Enrichment Analysis (GSEA) of DEGs was performed with the fgsea package in R. Genes were ranked based on the fold changes obtained with the treat method from the limma package. The enrichment score was calculated from the genesets of Epithelial cell differentiation markers and Hallmark Epithelial-Mesenchymal Transition from GSEA (http://www.gsea-msigdb.org/gsea/index.jsp).

Transcription Factor Enrichment Analysis (TFEA) based on DEGs was performed using the web-based tool ChIP-X Enrichment Analysis 3 **(**ChEA3**)** [https://maayanlab.cloud/chea3/ (34)]. TF enrichment scores were ranked using the MeanRank method because it performs the best in the ChEA3 benchmark.

### Statistical analysis

Unless stated otherwise, results are expressed as means ± SD. Statistical significance was calculated using a two-tailed unpaired or paired Student’s *t*-test. *P*-values< 0.05 were considered statistically significant. Outliers were removed using a Grubbs test.

The expression variation rate of each transcription factor during post-partum thymic regeneration compared to the expression value in non-pregnant females was evaluated by calculating the error sum of squares (SSE):


$$SSE={\sum_{i=1}^{n}\left(X_{i}-\bar{X}\right)}^{2}$$

## Results

### *Klf4* is highly expressed and regulated in TECs

**Figure 1:**
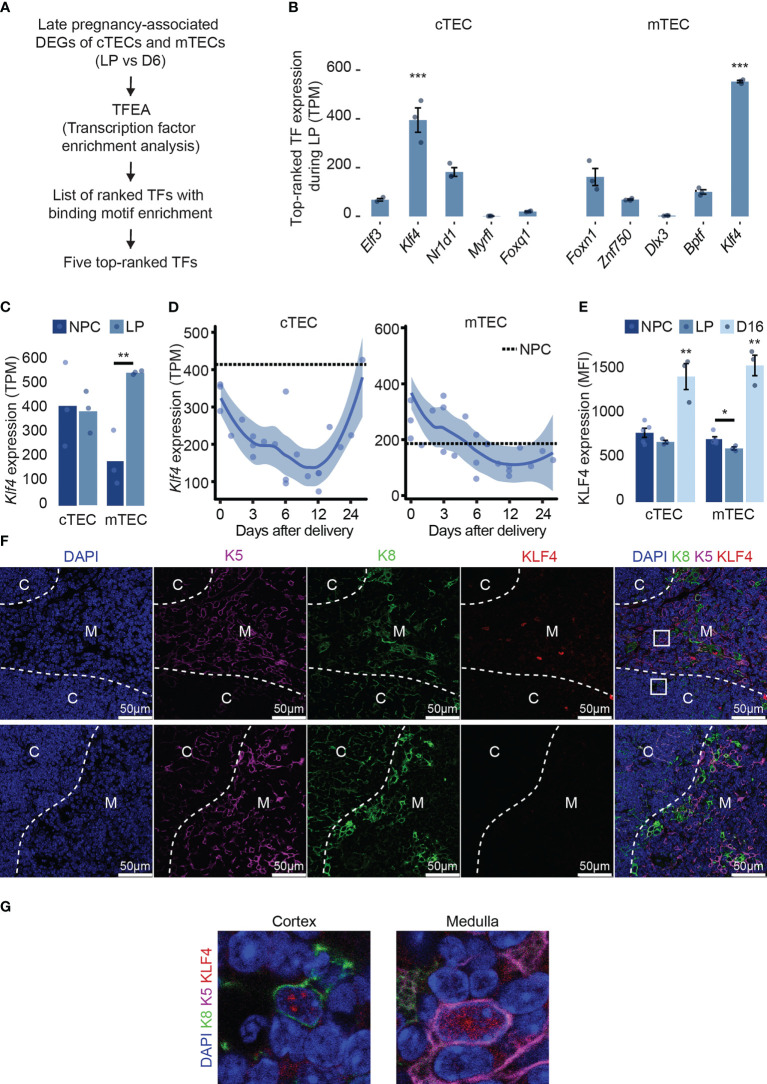
*Klf4* is highly expressed and regulated in TECs. **(A)** Pipeline selection for identifying TFs with binding motifs enrichments among genes differentially expressed at maximal thymic involution during late pregnancy (LP) compared to day 6 (D6) post-partum in both cTECs and mTECs ( (15), GSE138494). **(B)** Expression of five top-ranked TFs expression (TPM, transcripts per million) from TFEA analysis in both cTEC and mTEC during late pregnancy. TFs are ordered from the lowest to the highest MeanRank score on the barplot. Mean TPM was retrieved from previously published RNA-seq data (accession number GSE 138494). **(C)** *Klf4* expression (TPM) in cTECs and mTECs from non-pregnant controls (NPC) and during late pregnancy (LP). **(D)** *Klf4* expression (TPM) in cTECs and mTECs from delivery (D0) to D28 post-delivery. A dotted line represents NPC. **(E)** KLF4 expression (MFI) in cTECs and mTECs from NPC, pregnant (LP), and D16 post-partum females (D16). Significance was assessed using an unpaired two-tailed Student’s *t*-test. (*p<0.05 and **p<0.01). **(F)** KLF4 immunodetection on thymus section from LOX non-pregnant females. The sections were stained with anti-K5 (magenta), anti-K8 (green), and anti-KLF4 (red) antibodies. Nuclei were stained with DAPI (blue). White arrows point to KLF4^+^K5^+^, KLF4^+^K8^+^, or KLF4^+^K8^+^K5^+^ cells. Images are representative of three mice and were taken using a ×40 objective. The bottom line shows control images obtained by replacing the anti-KLF4 antibody with the corresponding isotype. Dotted white lines delineate cortical **(C)**, the cortico-medullar junction (CMJ), and medullary (M) regions; scale bars, 50 μm. **(G)** Enlarged boxed areas from the cortex and the medulla display the nuclear localization of KLF4 in cTECs and mTECs. Images were taken with a 63x objective.

We previously reported that qualitative changes in cTECs drive pregnancy-associated thymic involution and post-partum regeneration (15). To identify TFs regulating gene expression in TECs during and after pregnancy, we used the TF prediction tool ChEA3 (34). We searched for enrichment of TF binding motifs in genes differentially expressed at two-time points: the end of gestation (16-18 days) and D6 post-parturition (**Figure 1A**). Among the five top-ranked TFs with the lowest MeanRank score in each cell type (**Supplementary Table 2**), we found enrichment for motifs recognized mostly by regulators of epithelial cell differentiation: KLF4, ELF3, FOXQ1, DLX3, FOXN1, and ZNF750 (24, 35–39). KLF4 binding motifs were enriched in pregnancy-associated DEGs of both cTECs and mTECs. Furthermore, *Klf4* transcripts showed the highest expression of the five top-ranked TFs for both cTECs and mTECs (**Figure 1B**; **Supplementary Table 2**). Expression of *Klf4* showed the most considerable variations between the end of gestation and D28 post-delivery in cTECs (**Supplementary Table 2**).

As TECs are a highly heterogeneous population, we asked whether a single subtype of TECs highly expresses *Klf4*. Using publicly available scRNA-seq data (31) of TECs from 16 weeks aged females, we analyzed *Klf4* expression in different TEC subtypes. Although very few cells were available for rare TEC subsets (e.g., 11 cells for tuft-like mTEC), we found that a proportion of at least 35% of cells expressed *Klf4* (**Supplementary Figure 1A**). Previous reports have emphasized the importance of *Klf4* for epithelial homeostasis in many tissues like the lung, skin, and intestine using a conditional deletion (19, 20, 22). We found that *Klf4* was expressed at similar or higher levels in TECs relative to other epithelial cells (e.g., skin, colon, and lung) (**Supplementary Figure 1B**). During late pregnancy, *Klf4* expression was normal in cTECs but upregulated in mTECs (**Figure 1C**). In both TEC subsets, *Klf4* expression severely decreased after delivery and increased sharply in cTECs during late regeneration (D16 to D28 post-partum, **Figure 1D**). TFs typically have low protein stability, allowing rapid transitions of cell status. However, KLF4 is a highly stable protein (half-life > 24h), leading to potential discrepancies between mRNA and protein levels (40). Thus, we measured KLF4 protein expression in TECs from non-pregnant, pregnant (end of gestation), and D16 post-partum females using flow cytometry (**Figure 1E**). In cTECs, KLF4 expression was similar in late pregnancy and age-matched non-pregnant females. In mTECs, KLF4 expression decreased slightly compared to non-pregnant females. However, KLF4 expression was significantly higher during late regeneration (D16) in both cTECs and mTECs (**Figure 1E**). The discrepancy between mRNA and protein expression of *Klf4* shows that while *Klf4* transcription decreases during post-partum regeneration, KLF4 stability is higher compared to the end of gestation. Indeed, KLF4 stability is context-dependent, controlled by protein-protein interactions and post-transcriptional modifications (40). Interestingly, KLF4 plays different roles as a function of its stability (40), suggesting that KLF4 may exhibit different functions during thymic involution and regeneration. Using immunofluorescence on thymus sections, we confirmed the nuclear localization of KLF4 in K8^+^ cTECs and K5^+^ mTECs in the thymus of non-pregnant females (**Figures 1F, G**), as well as at the end of pregnancy and D16 following parturition (data not shown). Hence, *Klf4* expression in TECs is highly expressed at both mRNA and protein levels and is tightly regulated after delivery.

### *Klf4* deletion causes minor phenotypic modifications in the thymus

**Figure 2:**
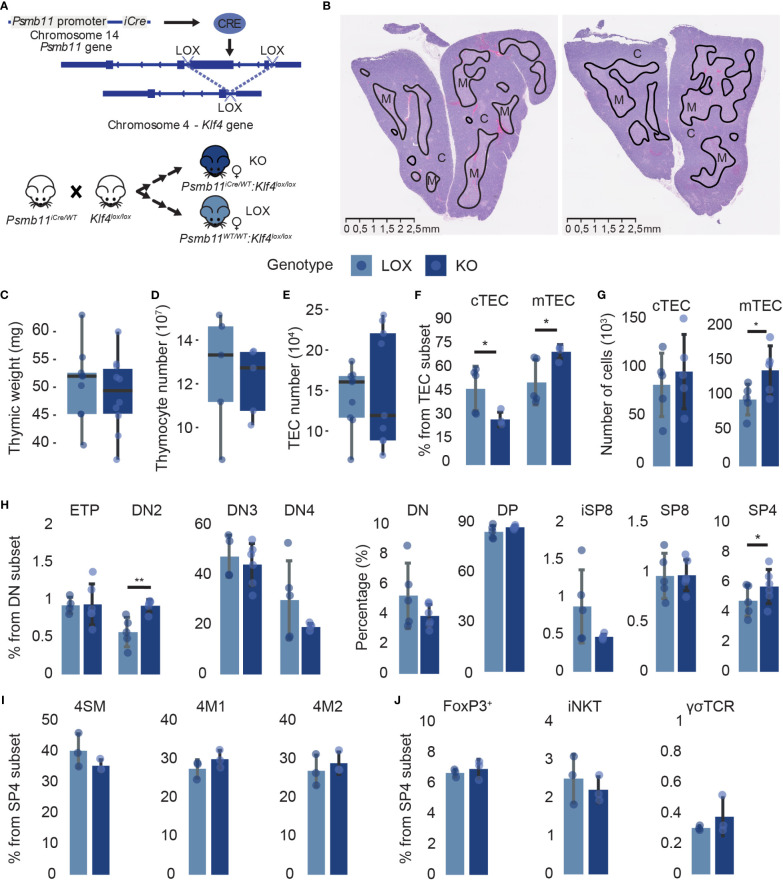
*Klf4* deletion shows a minor impact on thymic cellularity in homeostatic conditions. **(A)** Schematic representation of the Cre/loxP system used in the study. **(B)** H&E staining of thymus from non-pregnant LOX (left) and KO (right) females. Solid black lines delineate cortical **(C)** and medullary (M) regions. **(C–E)** Thymic weight (mg) **(C)**, thymocyte numbers **(D)**, and TEC numbers **(E)** in LOX and KO females (from n=5-10 mice per genotype). Results are expressed as the median and interquartile range (IQR). **(F, G)** cTEC and mTEC proportions **(F)** and absolute numbers **(G)** in LOX and KO females (n=4-5). **(H)** Double-negative thymocyte subpopulations and global thymocyte populations proportions in LOX and KO females (n=4-6). **(I, J)** Conventional **(I)** and unconventional **(J)** SP CD4 thymocyte proportions in LOX and KO females (n=3). CD1d tetramers, as well as Abs against CD25 and TCRγδ, were used to exclude NKT cells, regulatory T cells, and γδ T cells in the analysis of 4SM, 4M1, and 4M2 subsets. SM, semi-mature; M1, mature 1; M2, mature 2. TEC and thymocytes were analyzed in three and two independent experiments, respectively. LOX and KO genotypes are displayed in pale blue and dark blue, respectively. Significance was assessed using an unpaired two-tailed Student’s *t*-test. (*p<0.05 and **p<0.01).

To assess the role of *Klf4* in TECs, we crossed *Klf4^lox/lox^* female (28) and *Psmb11-iCre* male mice (29) (**Figure 2A**). *Psmb11* encodes for a thymoproteasome catalytic subunit solely expressed in TEC progenitors and cTECs (29). This model abolishes *Klf4* expression in cTECs and mTECs but not in other cell types, including thymocytes (**Supplementary Figures 1B, C**). Control females are littermates homozygous for the floxed *Klf4* allele and are referred to as LOX, while females also carrying the *Psmb11-iCre* are named KO. As homozygous mice for the Psmb11-iCre are deficient in functional PSMB11 protein and, consequently, show a defective generation of CD8+ T cells, we used *Psmb11^iCre/WT^* mice (29). *Klf4* deletion did not alter the thymic structure in 12-week-old female mice in homeostatic conditions. The boundary between the cortex and the medulla was well-defined, and the surface area occupied by the cortex and the medulla remained unchanged (**Figure 2B**). In addition, no significant differences in thymic weight or thymocyte and TEC numbers were observed between LOX and KO mice (**Figures 2C–E**). However, we observed minor variations in the proportion of TEC and thymocyte subsets. Thus, the proportion of cTECs decreased while that of mTECs increased in KO mice (**Figure 2F**). These relative changes correlated with an increase in the absolute number of mTECs in KO (**Figure 2G**). KO mice also showed a slight increase in the proportion of DN2 thymocytes and a commensurate decrease in DN3 and DN4 thymocytes (**Figure 2H**). The proportion and number of CD4 SP thymocytes were significantly increased in KO mice (**Figure 2H**; **Supplementary Figure 2B**). Frequencies of conventional (semi-mature, mature 1, and mature 2, (41, 42)) and unconventional (iNKT, Foxp3+ Tregs, and TCRγδ) CD4 SP thymocytes remained unchanged, suggesting a global CD4 SP-biased selection (**Figures 2I, J**). Overall, *Klf4* deletion in TEC showed no impact on the thymic structure and global cellularity in homeostatic conditions and only slightly affected the cTEC-to-mTEC ratio and the DN2 and CD4 SP thymocyte subsets.

**Figure 3:**
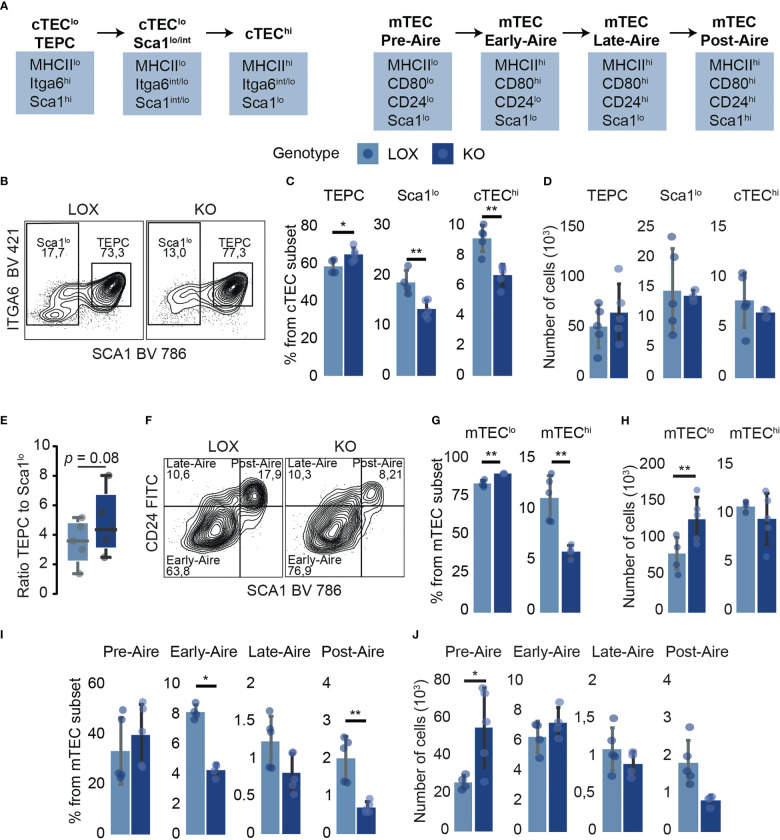
TEC subpopulation analysis suggests a role for *Klf4* in maintaining TEC differentiation in homeostatic conditions. **(A)** Schematic model representing markers used for TEC subpopulation identification (41, 42). **(B)** Flow cytometry contour plots representing Sca1^lo^ and TEPC proportions in LOX and KO females. **(C, D)** Proportions **(C)** and absolute numbers **(D)** of cTEC subpopulations (from n=4-5 mice per genotype). **(E)** The ratio of TEPC to Sca1^lo^ absolute numbers in KO and LOX females (n=4-5). Results are expressed as the median and interquartile range (IQR). **(F)** Flow cytometry contour plots representing Early-, Late-, and Post-Aire proportions in LOX and KO females. **(G, H)** Proportions **(G)** and absolute numbers **(H)** of mTEC^lo^ and mTEC^hi^ in LOX and KO females. **(I, J)** Proportions **(I)** and absolute numbers **(J)** of mTEC subpopulations (n=4-5). All analyses were performed in thymi of 12 weeks aged non-pregnant females. TECs were tested in three independent experiments, respectively. LOX and KO genotypes are displayed in pale blue and dark blue, respectively. Significance was assessed using an unpaired two-tailed Student’s *t*-test. (*p<0.05 and **p<0.01).

We further investigated the impact of *Klf4* deletion in discrete TEC subpopulations. cTECs and mTECs can be subdivided into subsets representing their maturation stages based on MHCII, Sca1, and α6-integrin expression for cTECs (41) and MHCII, CD24, and Sca1 expression for mTECs (42) (**Figure 3A**; **Supplementary Figure 2C**). Inter-sample variations affect absolute cell numbers more than cell proportions (43, 44). Therefore, we focused on percentages rather than absolute numbers. Despite slight proportional differences, *Klf4* deletion significantly increased thymic epithelial progenitor cells (TEPCs) and concomitantly reduced Sca1^lo^ and cTEC^hi^ subsets (**Figures 3B–E**). The proliferation and apoptosis in TEC subsets were analyzed using *in vivo* EdU incorporation and Annexin V (AnV) staining, respectively. *Ex vivo* studies of TEC apoptosis are challenging to interpret as thymus digestion induces high levels of Annexin V labeling (45). However, samples from KO and LOX females were treated similarly to minimize this bias. EdU and AnV analyses showed no proliferative and apoptotic modification in any cTEC subset (**Supplementary Figures 2D, E**), suggesting that TEPCs increased proportionally because of impaired cTEC differentiation in the absence of KLF4. Additionally, early-Aire mature and terminally differentiated post-Aire mTEC subsets were less abundant in KO females relative to LOX females (**Figures 3F–J**). Surprisingly, the proportion of proliferative cells slightly increased in mature late- and post-Aire mTEC from KO females, despite a numerical decrease in these populations (**Figure 2J**; **Supplementary Figure 2F**). Similar to cTECs, there was no impact of *Klf4* deletion on the proportion of apoptotic mTECs (**Supplementary Figure 2G**). Thus, the increase of mature mTEC proliferation might compensate for the reduction of *de novo* differentiation. These results support a role for *Klf4* in maintaining both cTEC and mTEC differentiation in homeostatic conditions.

### *Klf4* deletion leads to a cell-type specific downregulation of TEC terminal differentiation markers

**Figure 4:**
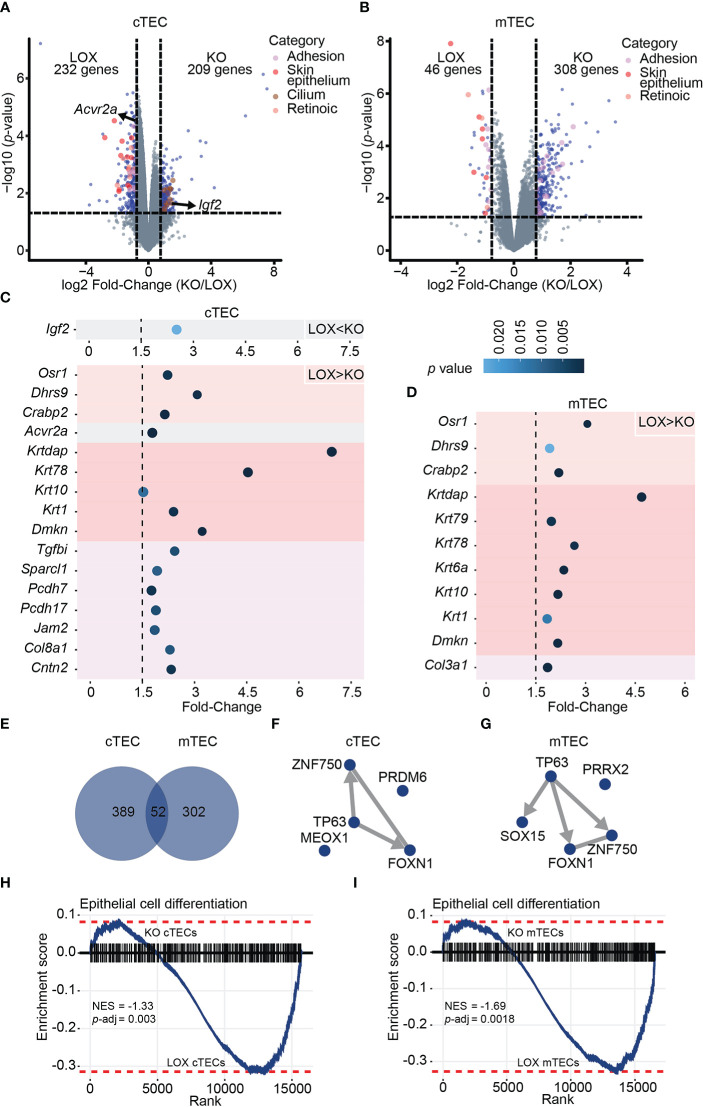
*Klf4* deletion leads to a cell-type-specific downregulation of terminal differentiation markers in TECs from non-pregnant females. **(A, B)** Volcano plots depicting differential gene expression in cTECs **(A)** and mTECs **(B)** between LOX and KO non-pregnant females. Colored dots represent DEGs with a *p*-value< 0.05 and a fold-change > 1.5. **(C, D)** Fold-Change of selected DEGs in cTECs **(C)** and mTECs **(D)** between LOX and KO females. The color circle gradient correlates with the *p*-value. **(E)** Venn diagram showing the overlap between DEGs identified in cTECs and mTECs of non-pregnant females. **(F, G)** TF-TF co-regulatory networks depict the interaction between TFs with the highest enrichment score based on the genes differentially expressed in cTECs **(F)** and mTECs **(G)** of LOX versus KO non-pregnant females. Edges are directed when ChIP-seq evidence supports the interaction and are undirected in the case of co-occurrence or co-expression evidence only. **(H, I)** Gene Set Enrichment Analysis (GSEA) for epithelial cell differentiation markers in cTEC **(H)** and mTEC **(I)** from LOX versus KO non-pregnant females. NES, normalized enrichment score; *p*-adj, false discovery rate adjusted *p*-value.

Given the modifications in TEC subsets observed in KO mice, we assessed the overall impact of *Klf4* on the transcriptome of cTECs and mTECs from 12-week-old KO and LOX female mice. Overall, the total number of DEGs was similar in cTECs and mTECs. KO cTECs displayed similar numbers of down- and upregulated DEGs (**Figure 4A**). In KO mTECs, most DEGs were upregulated (**Figure 4B**), suggesting that *Klf4* acted mainly as a transcriptional repressor. Keratin expression regulation is tightly linked to the epithelial cell differentiation stage, with differentiated epithelial cells expressing high molecular weight keratins (46). A group of high molecular weight keratins such as *Krt1*, *Krt2*, *Krt6*, and *Krt10* was downregulated in both cTECs and mTECs from KO females (**Figures 4A–D**; **Supplementary Table 3**). Interestingly, terminally differentiated mTECs in Hassall’s corpuscles highly express these specific keratins (42, 47). This low expression of keratins in the absence of *Klf4* suggests that *Klf4* regulates TEC differentiation. Thymic mesenchymal cells produce retinoic acid leading to TEC differentiation induction (48). TECs unable to respond to retinoic acid show an increase in proliferation and an accumulation of TEPCs (48). Interestingly, genes involved in the retinoic pathway, such as *Crabp2*, *Dhrs9*, and *Osr1*, decreased in the absence of *Klf4* in both cTECs and mTECs (**Figures 4A–D**; **Supplementary Table 3**). In addition, *Acvr2a*, recently described as a cTEC differentiation effector (49), was downregulated in cTECs from KO females (**Figures 4A, C**; **Supplementary Table 3**). Genes coding for cilium-associated proteins were upregulated in KO cTECs, suggesting a repressor role for *Klf4* (**Figures 4A, C**; **Supplementary Table 3**). A thymic ciliated cell population has been described by several groups (50–52). Although the function of ciliated thymic cells is unclear, we hypothesize that *Klf4* may be involved in regulating the composition of the thymic cortex by reducing the number of these ciliated thymic cells. We found that groups of extracellular matrix (ECM)- related genes such as tight junction proteins, ECM proteins, and collagens were decreased in KO cTECs (**Figures 4A, C**; **Supplementary Table 3**). Cell adhesion in the thymus is essential for its structure, TEC-thymocyte cross-talk, and thymocyte migration, suggesting that *Klf4* has a role in the stromal organization (53–55). *Klf4* is known to regulate cell adhesion in many tissues, and *Klf4* inhibition can alter cell migration in specific cancers (20, 56–58). *Igf2*, implicated in TEC growth and proliferation (59), was overexpressed in cTECs from KO females (**Figures 4A, C**; **Supplementary Table 3**). Interestingly, *Igf2* expression was associated with increased CD4 SP thymocytes (59). This might explain why we observed increased CD4 SP thymocytes in KO females. cTECs and mTECs shared only 7% (52/743) of DEGs, showing that KLF4 target genes are cell-type specific (**Figure 4E**).

KLF4 reorganizes chromatin and facilitates access to transcription factors for gene transcription (60). The differential activity of a given TF depends on the chromatin structure and collaborating factors, which could explain the cell-type specificity of *Klf4* in TECs (61). Therefore, we analyzed the enrichment of TF binding motifs among genes overexpressed in KO mice compared to LOX mice. Downregulated DEGs showed enrichment for motifs specific to FOXN1, TP63, and ZNF750 (**Figures 4F, G**; **Supplementary Table 4**). ZNF750 controls epithelial homeostasis by inhibiting progenitor genes while inducing differentiation genes (35). FOXN1 is a well-known key transcription factor for TEC differentiation (36, 62). TP63 blocks TEC proliferation and is essential for the development of the thymic epithelium, and the p63-FoxN1 regulatory axis regulates postnatal TEC homeostasis (63–66). Besides, both ZNF750 and FOXN1 are TP63 gene targets based on ChEA3 databases, and *Klf4* is a ZNF750 target gene in skin ECs (35). Hence, genes under expressed in KO TECs show a strong enrichment for binding motifs of TF regulating both cTEC and mTEC differentiation. Lastly, we performed a gene set enrichment analysis (GSEA) on genes under expressed in TEC from KO females using a list of epithelial cell differentiation markers. This analysis confirmed that a differentiation signature was significantly stronger in *Klf4*-expressing TECs (**Figures 4H, I**). Notwithstanding the absence of changes in structure and cellularity in thymi lacking *Klf4*, we conclude that *Klf4* maintains TEC differentiation at the phenotypic and transcriptomic levels.

### *Klf4* deletion severely aggravates thymic atrophy during late pregnancy

Recent studies showed that in several tissues, homeostasis is only marginally perturbed in the absence of *Klf4* (26). However, tissue disruption is much more pronounced when a stressor is applied to *Klf4*-deficient epithelial cells (toxins, mutations, etc.) than tissues with normal *Klf4* expression. In this way, *Klf4* may act as a critical “cell stability molecule” and an essential maintainer of tissue homeostasis (26). Pregnancy can be seen as an acute perturbation of the thymus. Thus, we decided to investigate how the deletion of KLF4 in TECs would affect thymic cell populations during late pregnancy when thymic involution is most severe.

**Figure 5:**
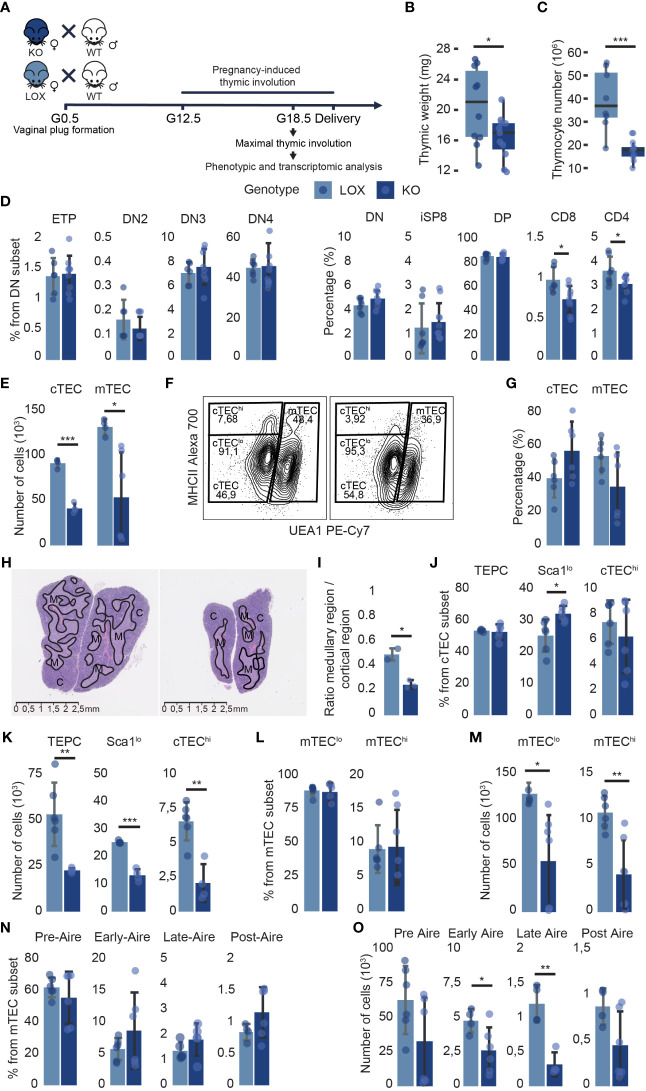
*Klf4* deletion in TECs alters thymus composition during pregnancy-induced thymic involution. **(A)** Experimental design to analyze the impact of *Klf4* deletion during late pregnancy. G0.5, G12.5, G18.5, gestational stages. **(B, C)** Thymic weight (mg) (from n=18-22 mice per genotype, tested in five independent experiments) **(B)**, and thymocyte number (n=8-10) **(C)** of pregnant LOX and KO females. Results are expressed as the median and IQR. **(D)** Proportions of thymocyte subsets in pregnant LOX and KO females (n=6-10 per group). **(E)** cTEC and mTEC numbers in pregnant LOX and KO females (n=4-6 per group). **(F)** Contour plots from flow cytometry analysis representing global cTEC and mTEC gates, and cTEC^hi^ and cTEC^lo^ gates within EpCAM^+^ CD45^-^ subsets in LOX (left) and KO (right) females. **(G)** cTEC and mTEC proportions of LOX and KO females (n=6). **(H)** H&E staining of thymus from pregnant LOX (left) and KO (right) females. Solid black lines delineate cortical **(C)** and medullary (M) regions. **(I)** Relative surface area of the medullary region compared to the cortical region per lobe of pregnant LOX and KO females. **(J, K)** cTEC subpopulation proportions **(J)** and absolute numbers **(K)** in pregnant LOX and KO females (n=4-6). **(L, M)** mTEC^lo^ and mTEC^hi^ percentages **(L)** and numbers **(M)** in KO and LOX females (n=3-5). **(N, O)** mTEC subpopulation proportions **(N)** and absolute numbers **(O)** in pregnant LOX and KO females (n=4-6). All analyses were performed on the thymi of pregnant females. TECs and thymocytes were analyzed in two and three independent experiments, respectively. LOX and KO genotypes are displayed in pale blue and dark blue, respectively. Significance was assessed using an unpaired two-tailed Student’s *t*-test. (*p<0.05, **p>0.01 and ***p>0.001).

We mated KO and LOX females with C57BL/6J (WT) males and analyzed the maternal thymus at the end of gestation when thymic involution reaches a nadir (**Figure 5A**). Pregnancy-induced thymic involution was more severe in KO females. They presented a lower thymic weight and a 45% decrease in thymocyte numbers compared to LOX females (**Figures 5B, C**). This thymocyte loss in KO pregnant females was TEC-dependent as *Klf4* was solely deleted in TECs (**Supplementary Figures 1C, D**). The global thymocyte loss affected all subsets but was more severe for CD4 and CD8 SP thymocytes, whose proportion was decreased (**Figure 5D**; **Supplementary Figures 3A, B**). Notably, while no changes in TEC numbers were observed in WT pregnant mice (15), KO females showed a 55% and 60% decrease in cTEC and mTEC numbers, respectively (**Figure 5E**). Consistent with the particularly severe depletion of SP thymocytes, the proportion of mTECs decreased compared to cTECs (**Figures 5F, G**). Moreover, the volume of the medulla was significantly reduced in KO thymi relative to the cortical region (**Figures 5H, I**). Although the thymic size was decreased in KO females, the corticomedullary demarcation remained unaffected. All TEC subsets were depleted in KO mice, but the cTEC^hi^ subset was particularly sensitive to *Klf4* deletion (**Figures 5F, J–O**). We conclude that *Klf4* is essential for maintaining TEC cellularity during late pregnancy.

### *Klf4* is essential to maintain cTEC integrity during late pregnancy and post-partum thymic regeneration

**Figure 6:**
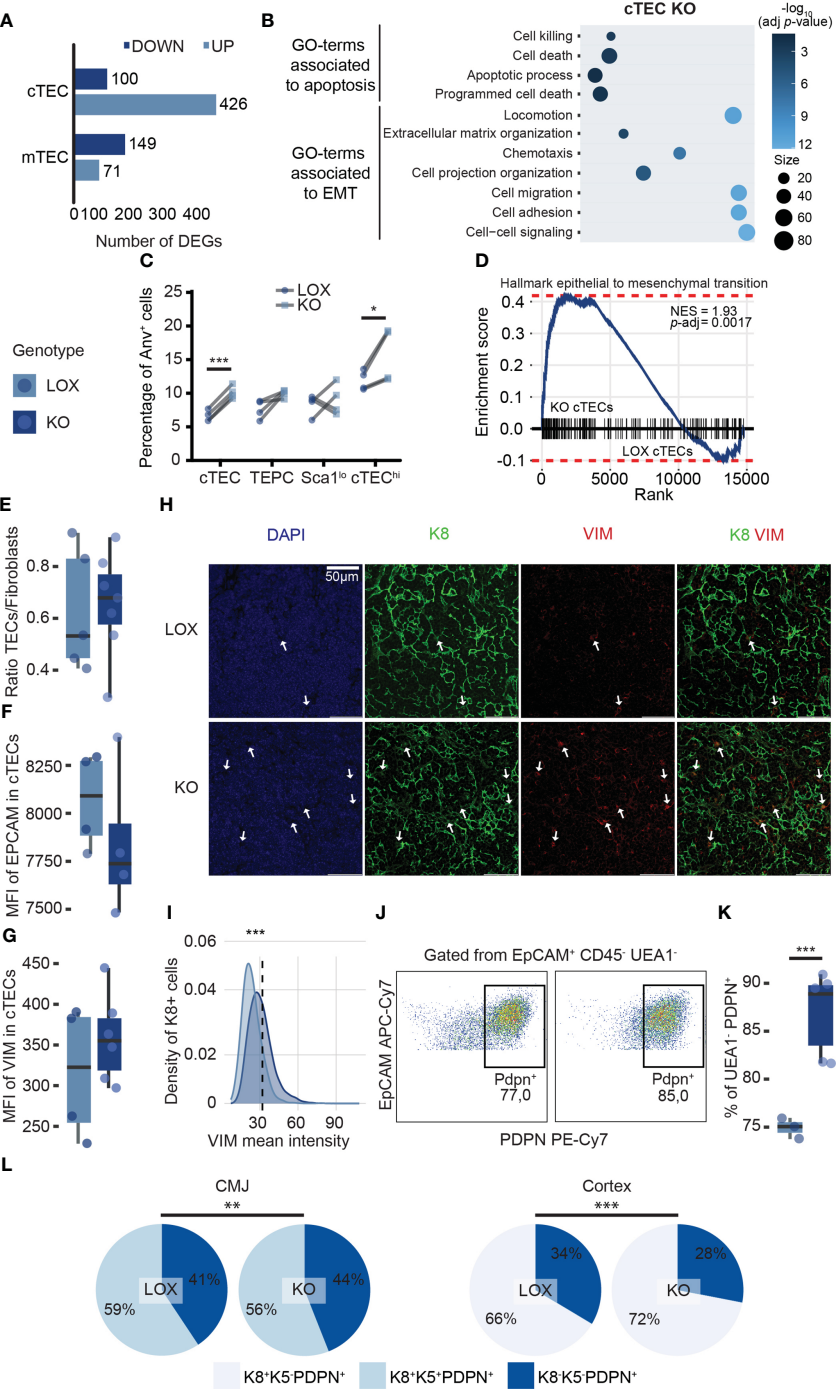
*Klf4* protects cTECs during late pregnancy by inhibiting apoptosis and EMP. **(A)** Number of DEGs upregulated (pale blue) or downregulated (dark blue) in cTECs and mTECs from LOX pregnant females compared to KO pregnant females. DEGs were identified with a *p*-value< 0.05 and a fold-change > 1.5. **(B)** Balloon plot depicting biological processes enriched in genes differentially expressed in LOX versus KO pregnant female. Only GO terms with an adjusted *p*-value< 0.05 are displayed (g:SCS threshold; Analysis performed with g:Profiler). **(C)** Paired plot showing the percentage of apoptotic cells in LOX and KO pregnant females. Significance was assessed using a paired Student’s *t*-test. (*p<0.05, ***p<0.001). **(D)** Gene Set Enrichment Analysis (GSEA) for epithelial to mesenchymal transition markers in cTEC from KO versus LOX pregnant females. NES, normalized enrichment score; *p*-adj, false discovery rate adjusted *p*-value. **(E)** Ratio TEC to fibroblasts in LOX and KO pregnant females. **(F, G)** Mean fluorescence intensity (MFI) of EpCAM **(F)** and VIM **(G)** in cTECs from LOX and KO pregnant females. **(H)** VIM immunodetection on cortex from LOX and KO pregnant thymus. The sections were stained with anti-K8 (green) and anti-VIM (red) antibodies. Nuclei were stained with DAPI (blue). Images are representative of two mice. A z-stack of ten 40X images of 0.346μm was turned into a single 2D image. Scale bar, 50 μm. **(I)** Density plot depicting the distribution of VIM mean cytometric intensity in K8+ cTECs from LOX and KO pregnant females. The dotted line represents the threshold for VIM intensity to classify VIM-negative and VIM-positive cells. Significance was assessed using a Pearson chi-square test (***p<0.001). **(J)** Dot plots from flow cytometry analysis representing PDPN^+^ gate within EpCAM^+^ CD45^-^ UEA1^-^ cells in LOX (left) and KO (right) females. **(K)** Percentage of UEA1^-^PDPN^+^ cells in LOX and KO pregnant females. LOX and KO genotypes are displayed in pale blue and dark blue, respectively. **(L)** Pie charts representing TEC subpopulation proportions according to K8, K5, and PDPN expression in the CMJ and the cortex region of LOX and KO pregnant females. Significance was assessed using a Pearson chi-square test (**p<0.01, ***p<0.001).

Five hundred twenty-six genes were differentially expressed, with 426 (81%) being upregulated in KO relative to LOX cTECs (**Figure 6A**). Only 220 genes were differentially expressed in mTECs, with 149 (68%) being down-regulated (**Figure 6A**). When we compared DEGs identified in steady-state conditions *vs*. pregnancy, we found that only 9.5% (127/1335) were shared (data not shown). This further illustrates that the effects of KLF4 are highly context-specific. Of note, no genes associated with ETP recruitment were downregulated in KO cTECs. GO terms analysis of the DEGs in KO cTECs during pregnancy showed enrichment for cell death (**Figure 6B**), which correlated with an increased proportion of apoptotic (AnV+) cTECs (mainly the cTEC^hi^ subset) in KO mice (**Figure 6C**). This is consistent with the fact that cTEC^hi^ cells are more sensitive than TEPCs and Sca1^lo^ cTECs to the loss of *Klf4* (**Figure 5K**). GO term analysis also showed that a large proportion of genes differentially expressed in KO cTECs were associated with an epithelial-to-mesenchymal transition (EMT) program (e.g., locomotion, extracellular matrix organization, cell adhesion, and migration) (**Figure 6B**). Indeed, *Klf4* maintains epithelial homeostasis by regulating EMT (e.g., corneal epithelium) and represents a potential target for pathologies partially caused by EMT (e.g., pulmonary fibrosis, cancer) (56–58, 67). KO cTECs are epithelial cells that express mesenchymal markers such as Vimentin (*Vim*), fibroblast growth factors (*Fgf11*, *Fgf21*), and *Ctnna2* (**Supplementary Table 3**). Gene Set Enrichment Analysis (GSEA) showed a significant EMT signature in KO cTECs compared to LOX cTECs (**Figure 6D**). To validate transcriptomic observations, we quantified thymic fibroblasts (tFbs) population in KO and LOX pregnant females based on EpCAM, CD45, PDPN, PDGFRαβ, CD31, and CD146 expression (68, 69) using flow cytometry. The ratio TECs to tFbs did not decrease in KO females (**Figure 6E**). However, flow cytometry analysis showed a downward trend of EpCAM expression, while VIM expression slightly increased in KO cTECs (**Figures 6F, G**). In addition, immunofluorescence analysis revealed that the intensity of VIM staining was significantly higher in KO K8^+^ cTECs. (**Figures 6H, I**; **Supplementary Figures 3C, D**). Hence, KO cTECs did not fully differentiate into tFbs but decreased epithelial features and increased mesenchymal characteristics. The ability of epithelial cells to express mixed epithelial/mesenchymal markers is called epithelial-to-mesenchymal plasticity (EMP) (70). EMP is widely observed and context-specific in various biological contexts, such as development and wound healing (70). In mTECs, GO terms analysis did not show enrichment for genes involved in cell proliferation or apoptosis that could explain their reduction (data not shown). This observation supports our previous conclusion that cTECs orchestrate thymic involution during pregnancy, while mTEC proliferation and differentiation mainly depend on thymocyte numbers (15, 18). Interestingly, we observed a significant increase of EpCAM^+^ CD45^-^ UEA1^-^ PDPN^+^ cells proportion in females lacking *Klf4* in TECs (**Figures 6J, K**). A UEA1^-^ PDPN^+^ TEC population described as inter-typical TECs or junctional TECs (jTECs) has previously been found to be localized in the CMJ (71, 72). Therefore, we investigated whether the UEA1^-^ PDPN^+^ cells were cTECs or jTECs. Using immunofluorescence, we quantified cell populations relative to the signal intensity of K8, K5, and PDPN staining in the CMJ and the thymic cortex of pregnant LOX and KO females (**Supplementary Figure 3E**). We found a conspicuous increase in the proportion of K8^+^PDPN^+^ cTECs in the thymic cortex of KO females compared to LOX females (**Figure 6L**). We also noted a minor decrease in K8^+^K5^+^PDPN^+^ TECs in the CMJ region (**Figure 6L**). We conclude that the expanded UEA1^-^PDPN^+^ TEC population observed by flow cytometry corresponds to cTECs undergoing EMP and not to jTECs. Overall, these data illuminate the non-redundant role of *Klf4* in maintaining thymic integrity during late pregnancy by inhibiting apoptosis and EMP in cTECs.

**Figure 7:**
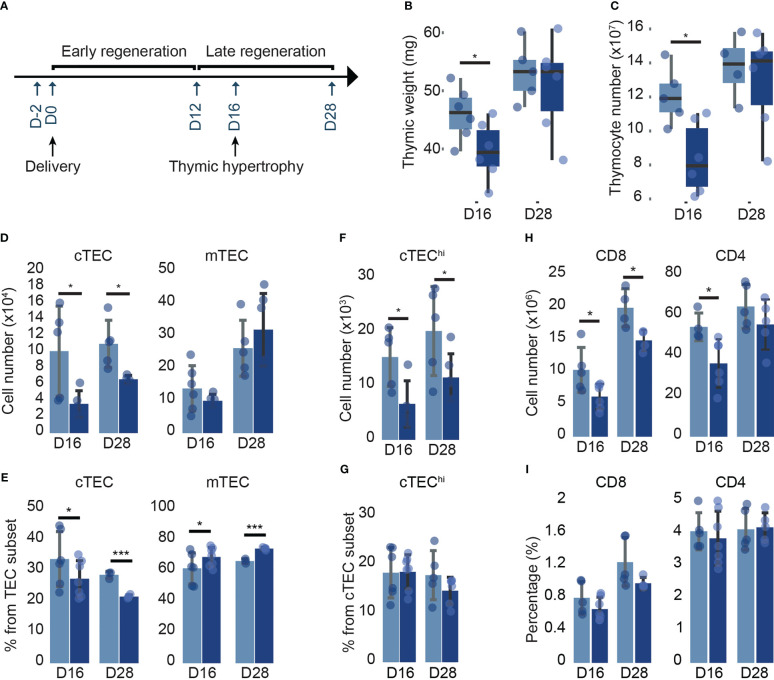
Profound modification in mature cTECs from KO females persists at D28 post-delivery. **(A)** Timeline of post-partum thymic regeneration. **(B, C)** Thymic weight (mg) **(B)** and thymocyte numbers **(C)** in LOX and KO females (from n=4-6 mice per genotype). **(D, E)** cTEC and mTEC numbers **(D)** and proportions **(E)** in LOX and KO females (n=5-7). **(F, G)** cTEC^hi^ number **(F)** and proportion **(G)** in LOX and KO females (n=4-5). **(H, I)** Simple-positive CD8 and CD4 thymocyte numbers **(H)** and proportions **(I)** in LOX and KO females (n=4-5). All analyses were performed in the thymi of females at D16, and D28 post-delivery tested in two independent experiments. LOX and KO genotypes are displayed in pale blue and dark blue, respectively. Significance was assessed using an unpaired two-tailed Student’s *t*-test. (*p<0.05).

We wondered whether the severe thymic involution in KO females would impact post-partum thymic regeneration. Therefore, we analyzed thymic growth during the late regeneration phase at D16 and D28 post-delivery (**Figure 7A**). We observed that thymic cellularity and weight were significantly decreased in KO females compared to LOX females at D16 post-delivery (**Figures 7B, C**). However, thymic cellularity and weight were restored at D28 post-delivery (**Figures 7B, C**). Interestingly, cTECs decreased by 60% and 40% after delivery in KO females at D16 and D28, respectively (**Figures 7D–G**). Likewise, SP CD4 and SP CD8 showed no alteration in proportions but a numerical decrease in KO females at D16 and D28 (**Figures 7H, I**). These results suggest that cTEC reduction affects thymocyte development during post-partum thymic regeneration in *Klf4*-deficient females. We conclude that *Klf4* deletion during pregnancy has a long-lasting effect on thymic regeneration.

## Discussion

During the lifespan of an individual, several factors can cause thymic atrophy. Infection, irradiation, and corticosteroids cause thymic atrophy by direct induction of thymocyte apoptosis (11, 71). In contrast, atrophy induced by sex hormones and age primarily affects TECs (11, 15). Progesterone-induced thymic involution during pregnancy has been proposed to be an energy-saving mechanism and to enhance maternal tolerance of the fetus (11, 16, 18). We found that KLF4 expression is high in TECs from non-pregnant and pregnant mice. While *Klf4* transcription drops abruptly following parturition, KLF4 protein is highly stable. The context-dependent stability of KLF4 suggests different functions during thymic involution and regeneration (40). Moreover, we noted that KLF4 binding motifs are significantly enriched in the DNA sequence of differentially expressed TEC genes at the end of pregnancy. The salient finding of our study is that deletion of *Klf4* in TECs has minimal effects under steady-state conditions but a significant impact during late pregnancy.

**Figure 8:**
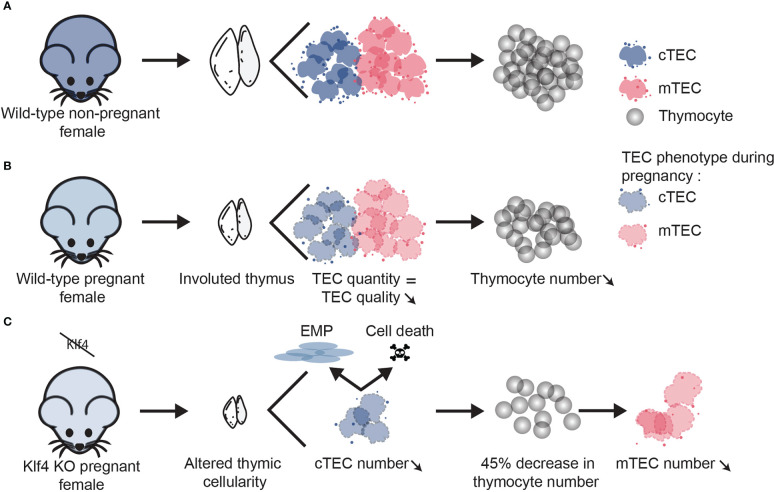
*Klf4* is a protective factor for thymus integrity during pregnancy-associated thymic involution. Working model depicting the role of *Klf4* in TEC homeostasis during late pregnancy. **(A)** Non-pregnant WT mice exhibit an age-appropriate level of TEC and thymocyte numbers. **(B)** During late pregnancy, thymocyte hypocellularity in WT mice is associated with functional defects in TECs without TEC loss. **(C)** When *Klf4* is deleted, cTEC apoptosis and EMP deplete the cTECs pool. As a result, thymocyte loss is aggravated, particularly for the SP thymocytes. Loss of SP thymocytes leads to a decrease in mTEC number.

In WT pregnant females, thymocyte development is paused due to altered cTEC function, but TEC numbers are unchanged (**Figures 8A, B**). In other epithelial tissues, *Klf4* has been described as a “cell stability molecule” that protects tissue homeostasis when a stressing agent is applied (26). In other words, KLF4 expression in epithelial cells appears important to protecting tissue integrity when an injury occurs. Indeed, *Klf4* deletion in injured tissues delays wound healing (21, 72). In line with this concept, we report that females lacking *Klf4* in TECs show significant alterations in thymic cellularity during pregnancy-induced thymic involution. These mice present a substantial reduction of both thymocytes and TEC numbers relative to WT pregnant females (**Figures 8B, C**). Transcriptomic and phenotypic analysis showed that *Klf4* maintains cTEC number during late pregnancy by inhibiting apoptosis and EMP abilities (**Figure 7C**). This is also coherent with reports of *Klf4* being an apoptotic and EMT suppressor in other types of epithelial cells (24, 56, 58, 67). In contrast to cTECs, the loss of mTECs in *Klf4*-deficient mice cannot be explained by mTEC-intrinsic transcriptomic changes. Cross-talk with SP thymocytes is essential for mTEC differentiation during embryonic development (73). Additionally, we previously showed a correlation between SP thymocyte expansion and mTEC proliferation during post-partum thymic regeneration (15). Consequently, we suggest that the loss of mTECs in KO pregnant females is caused by the depletion of SP thymocytes (**Figure 8C**).

These findings reveal that *Klf4* is a protective factor for maintaining cTEC numbers during the last trimester of pregnancy when thymic involution is paramount. Additional analyses would be required to determine whether the loss of KLF4 affects cTECs at earlier stages of pregnancy (when thymic involution is less pronounced). The significant thymic medulla atrophy in pregnant females lacking *Klf4* raises questions concerning fetal development. Indeed, RANK-expressing mTECs control the expansion of thymic Tregs, which are necessary for maternal tolerance to fetal alloantigens (74). Hence, in future studies, efforts should be made to quantify rates of miscarriage and splenic Treg numbers in *Klf4*-deficient mice. The striking pregnancy-induced thymic atrophy in *Klf4*-deficient mice had a long-lasting impact on mature cTECs and thymocyte development. Hence, further studies will be needed to determine whether delayed thymic regeneration impacts long-term immune function. In addition, this raises questions about the impact on maternal thymus integrity following repetitive pregnancies in *Klf4*-deficient females and the effectiveness of the protective role of KLF4 in older pregnant females.

Finally, this study highlights the potential interest in studying *Klf4* as a protective factor in other thymic injury models, such as glucocorticoid- or radiation-induced thymic involution. In particular, *Klf4* is a radioprotective factor in intestinal epithelial cells and may be a therapeutic target for TEC protection following irradiation treatment (25, 75, 76). A more complex issue will be to investigate whether upregulation of *Klf4* could prevent or reverse age-associated thymic involution. This is a pertinent question considering the impact of age-associated thymic atrophy on response to vaccines and the risk of infection, autoimmunity, and cancer (4, 6, 77).

## Data availability statement

The datasets presented in this study can be found in online repositories. The names of the repository/repositories and accession number(s) can be found below: https://www.ncbi.nlm.nih.gov/geo/, GSE 210885.

## Ethics statement

The animal study was reviewed and approved by Comité de Déontologie de l’Expérimentation sur les Animaux de l’Université de Montréal.

## Author contributions

The study design was performed by LD with the assistance of CP, MD-L, and SB. Data collection was performed by LD and SB with support from CH, VQ-HT, and CC. Bioinformatic analyses were performed by LD with the help of J-DL and VQ-HT. LD interpreted the data and wrote the first draft of the manuscript, revised by all authors. CP provided the financial and material resources necessary for the realization of this project. All authors contributed to the article and approved the submitted version.
