# Supplementary material for: Klf4 protects thymus integrity during late pregnancy
Source: Front Immunol. 2023 Apr 27;14:1016378. doi: 10.3389/fimmu.2023.1016378 (PMC10174329; doi:10.3389/fimmu.2023.1016378)
Supplement: Supplementary file 1 [file DataSheet_1.docx]

Supplementary Material

# Supplementary Figures and Tables

##
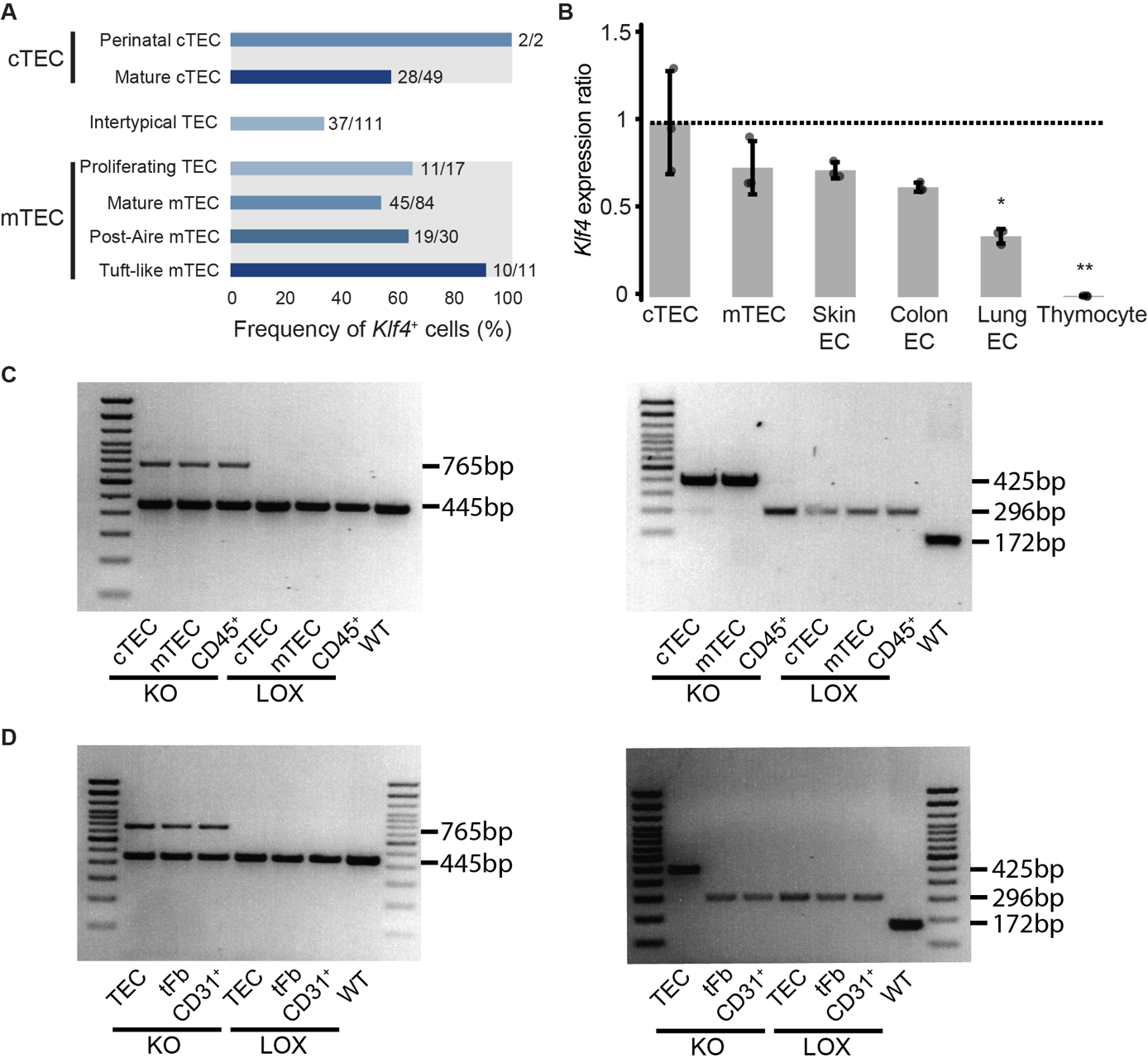
Supplementary Figures

**Supplementary Figure 1. (A)** Frequency of *Klf4^+^* cells in different TEC subtypes analyzed by scRNAseq (ArrayExpress : E-MTAB-8560, (1)). The number of *Klf4*^+^ cells on the total number for each subset is displayed on the bar graph. **(B)** *Klf4* expression ratio in epithelial cells from thymus, skin, colon, and lung of 12-week-old C57BL/6J mice (n=3). Thymocytes were used as non-stromal cell control. The *Klf4* expression ratio was calculated relative to its expression in cTEC as TPM. Results are expressed as mean ± SD. *P*-values are shown against cTECs and mTECs. Significance was assessed using a two-tailed Student *t*-test. (**p*<0.05 and ***p*<0.01). **(C, D)** PCR genotype analysis of DNA extracted from purified cTECs, mTECs, CD45^+^ cells (of which at least 95% are thymocytes) **(C)**, and purified TECs, tFbs, CD31^+^ cells **(D)** from KO and LOX females, to determine *Klf4* deletion efficiency and specificity in TECs. The PCR analysis for Psmb11-iCre (left panels) was performed using primers specific for iCre (765bp) or Psmb11 coding sequences (445bp) by Dr. Takahama’s group recommendations (2). PCR screening for *Klf4* (right panels) was performed using primers specific to the *wild-type Klf4* gene (172bp), floxed *Klf4* (296bp), and null *Klf4* (425bp) following the genotyping protocol of MMRRC (29877).


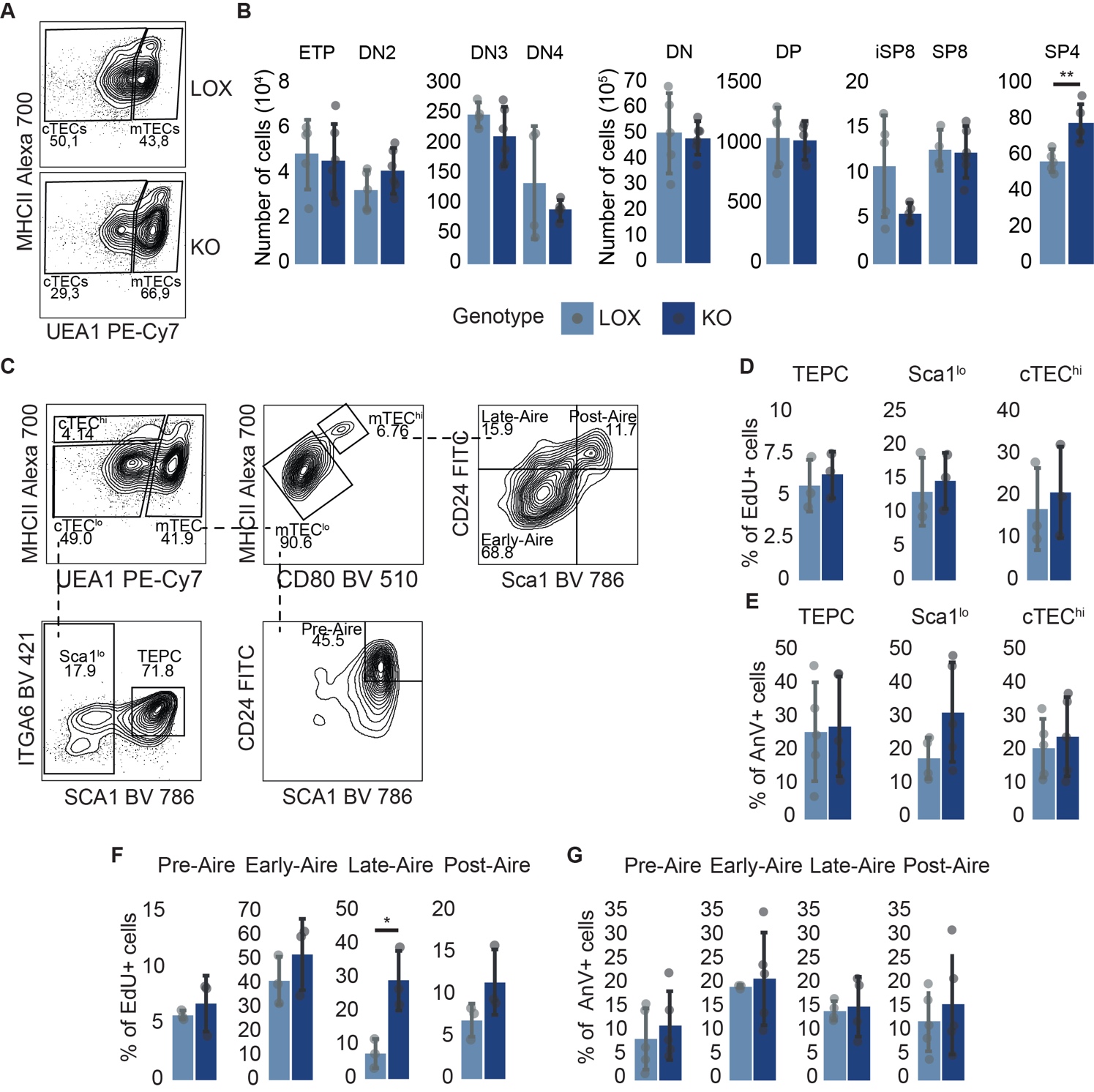


**Supplementary Figure 2.** **(A)** Flow cytometry contour plots representing cTECs and mTECs. **(B)** Absolute number of thymocyte subpopulations in KO and LOX females (n=4-6). **(C)** Gating strategy for analyzing cTEC and mTEC subpopulations by flow cytometry. **(D, E)** Proportion of proliferating EdU^+^ **(D)** and apoptotic AnV^+^ **(E)** cTEC subpopulations in KO and LOX mice (n=3-5). **(F, G)** Proportion of proliferating EdU^+^ **(F)** and apoptotic AnV^+^ **(G)** mTEC subpopulations in KO and LOX mice (n=3-5). KO and LOX genotypes are displayed in dark blue and pale blue, respectively. Significance was assessed using a two-tailed student *t*-test. (**p*<0.05)

**
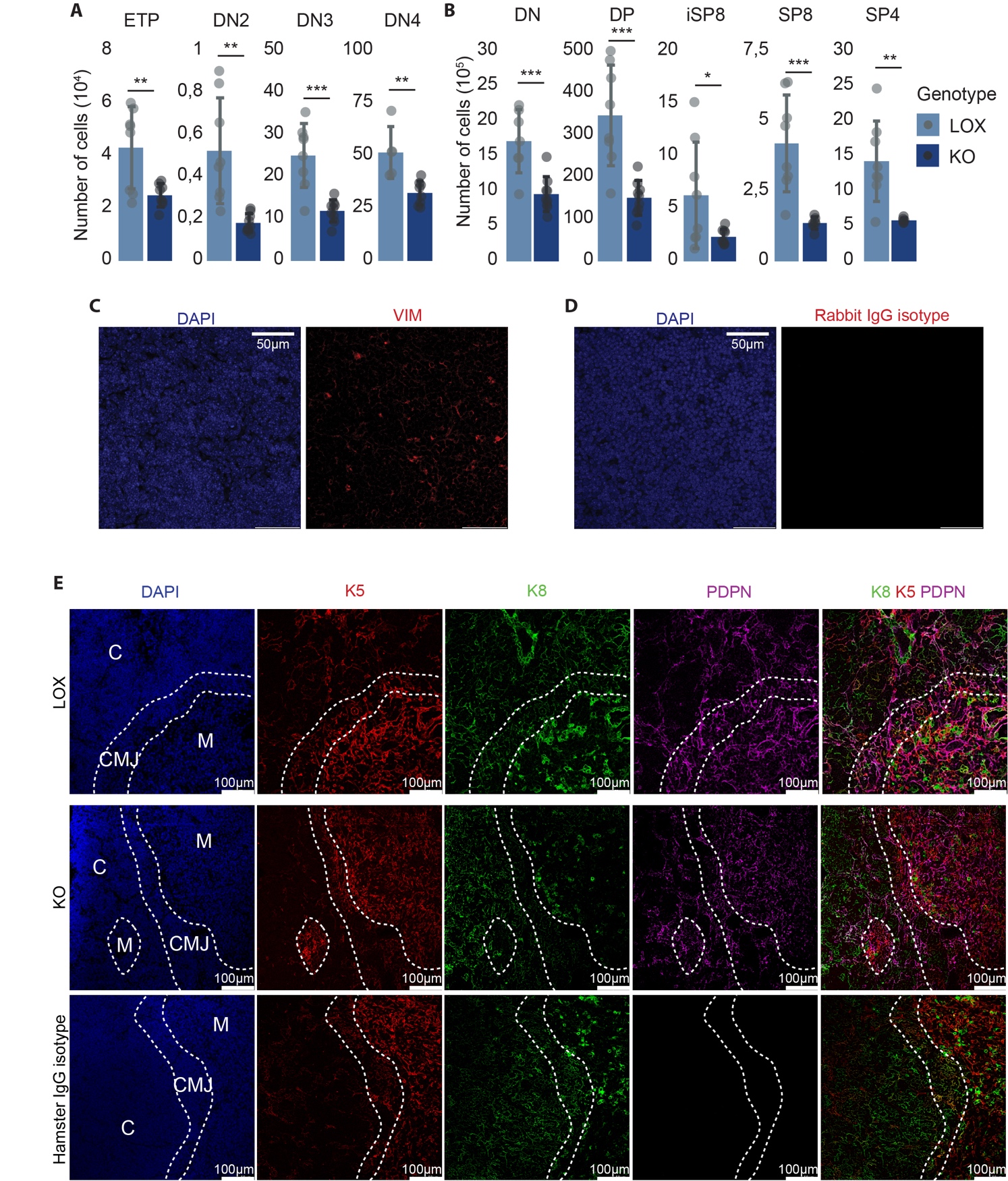
**

**Supplementary Figure 3. (A, B)** DN subsets **(A)** and global thymocyte subsets **(B)** in pregnant KO and LOX females (n=6-9). KO and LOX genotypes are displayed in dark blue and pale blue, respectively. Significance was assessed using a two-tailed Student *t*-test. (**p*<0.05, ***p*<0.01 and ****p*<0.001). **(C, D)** Sections of thymic cortex stained with anti-VIM antibody **(C)** or rabbit IgG isotype **(D)**. Nuclei were stained with DAPI (blue). Images are representative of 2 mice and were taken using a ×40 objective. Scale bar, 50μm. **(E)** Thymic sections were stained with anti-K5 (red), anti-K8 (green), and anti-PDPN (magenta) antibodies. The bottom panels show control staining by replacing anti-PDPN antibody with the corresponding isotype. Nuclei were stained with DAPI (blue). Dotted white lines delineate cortical (C), cortico-medullar junction (CMJ), and medullary (M) regions. Images are representative of 2 mice and were taken using a ×20 objective. Scale bar, 100μm.

## Supplementary Tables

**Supplementary Table 1.** Reagents used for flow cytometry (FC) analysis and immunofluorescence (IF) microscopy.

| Reagents | Catalog number | Clone | Supplier | Application |
| --- | --- | --- | --- | --- |
| Collagenase D | 11088866001 | - | Sigma | Tissue dissociation |
| DNase I | D25-1G | - | Sigma | Tissue dissociation |
| Papain | LS003119 | - | Worthington-Biochem | Tissue dissociation |
| LS Columns | 130-042-401 | - | Miltenyi Biotec | FC |
| CD326 (EpCAM) Microbeads mouse | 130-105-958 | - | Miltenyi Biotec | FC |
| EpCAM – APC-Cy7 | 118218 | G8.8 | BioLegend | FC |
| CD45 – PE-Cy5 | 553082 | 30-F11 | BD Biosciences | FC |
| CD45 – APC | 559864 | 30-F11 | BD Biosciences | FC |
| CD45 – Alexa Fluor 700 | 560510 | 30-F11 | BD Biosciences | FC |
| UEA1 – biotinylated | B-1065 | - | Vector Laboratories | FC |
| I-A/I-E – Alexa Fluor 700 | 107622 | M5/114.15.2 | BioLegend | FC |
| H-2K[b] – PE | 562832 | AF6-885 | BD Biosciences | FC |
| Streptavidin – PE-Cy7 | 557598 | - | BD Biosciences | FC |
| Streptavidin – BV421 | 405226 | - | Biolegend | FC |
| Propidium iodine | 51-66211E | - | BD Biosciences | FC |
| 7AAD | 559925 | - | BD Biosciences | FC |
| CD8a – PE-Cy5 | 553034 | 53-6.7 | BD Biosciences | FC |
| CD4 – APC-Cy7 | 552051 | GK1.5 | BD Biosciences | FC |
| TCRβ – FITC | 553171 | H57-597 | BD Biosciences | FC |
| cKit – PE-Cy7 | 558163 | 2B8 | BD Biosciences | FC |
| CD25 – APC | 557192 | PC61 | BD Biosciences | FC |
| CD44 – PE | 553134 | IM7 | BD Biosciences | FC |
| NK1.1 – biotinylated | 553163 | PKI36 | BD Biosciences | FC |
| Cd11c – biotinylated | 553800 | Hl3 | BD Biosciences | FC |
| TCRγ/δ – biotinylated | 118103 | GL3 | Biolegend | FC |
| Biotin mouse lineage panel | 133307 | - | Biolegend | FC |
| PE anti-mouse Ki67 set | 556027 | B56 | BD Biosciences | FC |
| Transcription factor staining buffer set | 00-5523-00 | - | Invitrogen | FC |
| Live/dead blue | L23105 | - | Thermofisher | FC |
| Ly6A/E – BV786 | 563991 | D7 | BD Biosciences | FC |
| Itga6 – BV421 | 313624 | GoH3 | Biolegend | FC |
| CD80 – BV510 | 104741 | 16-10A1 | Biolegend | FC |
| CD24 – FITC | 101806 | M1/69 | Biolegend | FC |
| Annexin V – PE | 556421 | - | BD Biosciences | FC |
| 5-Ethynyl-2’-deoxyuridine | 900584 | - | Sigma | In vivo analysis |
| Click-iT™ Plus EdU Alexa Fluor™ 647 Flow Cytometry Assay Kit | C10634 | - | Invitrogen | FC |
| Gp38 (Pdpn) – PE-Cy7 | 127412 | 8.1.1 | Biolegend | FC |
| PDGFRA – biotinylated | 13-1401-82 | APB5 | eBioscience | FC |
| PDGFRB – biotinylated | 13-1402-82 | APB5 | eBioscience | FC |
| CD146 – PE | 134703 | ME-9F1 | BioLegend | FC |
| CD31 – Alexa Fluor 488 | 102414 | 390 | BioLegend | FC |
| CD1d-PBS-57 tetramer – BV421 | 32730 | - | National Institutes of Health (NIH) | FC |
| CD8a – BV480 | 566096 | 53-6.7 | BD Biosciences | FC |
| CD44 – BV650 | 103049 | IM7 | BioLegend | FC |
| NK1.1 – FITC | 553164 | PKI36 | BD Biosciences | FC |
| Foxp3 – PE | 12-5773-80 | FJK-16s | eBiosciences | FC |
| TCRβ – PE/Dazzle594 | 109240 | H57-597 | BioLegend | FC |
| TCRγ/δ – APC | 118115 | GL3 | Biolegend | FC |
| TCRγ/δ – PE | 553176 | GL3 | BD Biosciences | FC |
| CD4 – Alexa Fluor 700 | 554656 | RM4-5 | BD Biosciences | FC |
| CD25 – APC-Cy7 | 102026 | PC61 | BioLegend | FC |
| H-2K[b] – APC | 17-5958-82 | AF6-88.5.53 | eBioscience | FC |
| CD69 – PE-Cy7 | 552879 | H1.2F3 | BD Biosciences | FC |
| CCR7 – PE-Cy5 | 120114 | 4B12 | eBioscience | FC |
| Klf4 – purified | PA5-27440 | Polyclonal rabbit | Invitrogen | FC |
| Donkey anti-rabbit – PE | 558416 | - | BD Biosciences | FC |
| Klf4 - purified | AF3158 | Polyclonal Goat | R&D Systems | IF |
| Bovine anti-goat–purified | 805-005-180 | - | Jackson ImmunoResearch | IF |
| Goat anti-bovine Alexa Fluor 594 | 101-585-003 | - | Jackson ImmunoResearch | IF |
| Podoplanin - purified | 14-5381-82 | 8.1.1 | Invitrogen | IF |
| Goat anti-hamster – Alexa Fluor 488 | A21110 | - | Invitrogen | IF |
| Vimentin- Alexa Fluor 594 | BS-0756R | Polyclonal | Bioss | FC - IF |
| Cytokeratin 8 - purified | MABT329M | TROMA-I | Sigma-Aldrich | IF |
| Chicken anti-rat – Alexa Fluor 488 | A21470 | - | Molecular Probes | IF |
| Goat anti-rat – Alexa Fluor 555 | A21434 | - | Invitrogen | IF |
| Keratin 5 polyclonal – purified | 905504 | Poly19055 | BioLegend | IF |
| Goat anti-rabbit – Alexa Fluor 680 | A21077 | - | Invitrogen | IF |

**Supplementary Table 2.** Five top-ranked TFs predicted to bind the DEGs in TECs at the end of gestation vs. D6 postpartum

|  | TF | TFEA | | Expression variation rate during PTR | |
| --- | --- | --- | --- | --- | --- |
|  |  | MeanRank ^a^ | Rank | SSE ^b^ | Rank |
| cTEC | *Elf3* | 6 | #1 | 0.35 | #4 |
|  | *Klf4* | 16.2 | #2 | 1.48 | #1 |
|  | *Nr1d1* | 20 | #3 | 0.68 | #2 |
|  | *Myrfl* | 25.5 | #4 | 0.16 | #5 |
|  | *Foxq1* | 26.33 | #5 | 0.62 | #3 |
| mTEC | *Foxn1* | 9.33 | #1 | 0.48 | #4 |
|  | *Znf750* | 20.25 | #2 | 0.29 | #5 |
|  | *Dlx3* | 32 | #3 | 1.60 | #1 |
|  | *Bptf* | 36 | #4 | 0.52 | #3 |
|  | *Klf4* | 36.6 | #5 | 0.57 | #2 |

^a^Transcription factor enrichment analysis (TFEA) using the tool ChEA3 (https://maayanlab.cloud/chea3/). MeanRank lower scores indicate more relevancy to the transcription factor.

^b^ Variation of the expression of each TF during postpartum thymic regeneration (PTR) relative to non-pregnant control. TPM expression was retrieved from previously published RNA-seq data (accession number GSE 138494). The equation used to calculate SSE is described in the “Materials and Methods” section.

**Supplementary Table 3.** Differentially expressed genes (DEGs) in both cTECs and mTECs between KO versus LOX pregnant and non-pregnant females. (Excel file)

**Supplementary Table 4.** Five top-ranked TFs predicted to bind genes underexpressed in *Klf4*-deficient non-pregnant females.

|  | TF | MeanRank ^a^ |
| --- | --- | --- |
| cTEC | *Znf750* | 30 |
|  | *Tp63* | 39.4 |
|  | *Prdm6* | 44.67 |
|  | *Foxn1* | 46.33 |
|  | *Meox1* | 50 |
| mTEC | *Znf750* | 5 |
|  | *Foxn1* | 11 |
|  | *Tp63* | 29.8 |
|  | *Sox15* | 45.33 |
|  | *Prrx2* | 48.33 |

^a^Transcription factor enrichment analysis (TFEA) using the tool ChEA3 (https://maayanlab.cloud/chea3/). MeanRank lower scores indicate more relevancy to the transcription factor.

1. Baran-Gale J, Morgan MD, Maio S, Dhalla F, Calvo-Asensio I, Deadman ME, Handel AE, Maynard A, Chen S, Green F, et al. Ageing compromises mouse thymus function and remodels epithelial cell differentiation. *eLife* (2020) 9:e56221. doi: 10.7554/eLife.56221

2. Ohigashi I, Zuklys S, Sakata M, Mayer CE, Zhanybekova S, Murata S, Tanaka K, Holländer GA, Takahama Y. Aire-expressing thymic medullary epithelial cells originate from β5t-expressing progenitor cells. *Proc Natl Acad Sci U S A* (2013) 110:9885–9890. doi: 10.1073/pnas.1301799110
